# Internal construct validity of the stress-energy questionnaire in a working population, a cohort study

**DOI:** 10.1186/s12889-015-1524-9

**Published:** 2015-02-25

**Authors:** Emina Hadzibajramovic, Gunnar Ahlborg, Anna Grimby-Ekman, Åsa Lundgren-Nilsson

**Affiliations:** The Institute of Stress Medicine, Region Västra Götaland, Gothenburg, Sweden; Department of Public Health and Community Medicine, Sahlgrenska Academy, University of Gothenburg, Gothenburg, Sweden; Institute of Neuroscience and Physiology, Department of Clinical Neuroscience and Rehabilitation, Sahlgrenska Academy, University of Gothenburg, Gothenburg, Sweden

**Keywords:** Stress-energy questionnaire, Work stress, Rasch analysis, Questionnaires, Ordinal data

## Abstract

**Background:**

Psychosocial stress at work has been recognised as one of the most important factors behind the increase in sick leave due to stress-related mental disorders. It is therefore important to be able to measure perceived work stress in a way that is both valid and reliable. It has been suggested that the Stress-Energy Questionnaire (SEQ) could be a useful tool for measuring mood (stress and energy) at work and it has been used in many Scandinavian studies. The aim of the study is to examine the internal construct validity of the SEQ in a working population and to address measurement issues, such as the ordering of response categories and potential differences in how women and men use the scale – what is termed differential item functioning (DIF).

**Methods:**

The data used in the present study is baseline data from a longitudinal cohort study aimed at evaluating psychosocial working conditions, stress, health and well-being among employees in two human service organisations in Western Sweden. A modern psychometric approach for scale validations, the Rasch model, was used.

**Results:**

Stress items showed a satisfactory fit to the model. Problems related to unidimensionality and local dependence were found when the six stress items were fitted to the model, but these could be resolved by using two testlets. As regards the energy scale, although the final analysis showed an acceptable fit to the model some scale problems were identified. The item *dull* had disordered thresholds and DIF for gender was detected for the item *passive*. The items were not well targeted to the persons, with skewness towards high energy. This might explain the scale problems that were detected but these problems need to be investigated in a group where the level of energy is spread across the trait, measured by the SEQ.

**Conclusion:**

The stress scale of the SEQ has good psychometric properties and provides a useful tool for assessing work-related stress, on both group and individual levels. However, the limitations of the energy scale make it suitable for group evaluations only. The energy scale needs to be evaluated further in different settings and populations.

## Background

Work-related stress and sick leave due to stress-related mental disorders was acknowledged as an increasing cause for concern throughout the EU during the 1990s [1]. In a recent report by the European Commission, stress is highlighted as one of the psychosocial risk factors that are a source of growing unease in occupational health [1]. Psychosocial stress at work was found to be one of the most important factors behind this increase [2-4]. The effects of prolonged exposure to stress can have serious consequences, not only for individuals but also for workplaces and society in terms of reduced work performance and increased long-term sick leave as a result of mental health problems [1,5,6]. It is therefore crucial to prevent, eliminate and reduce problems caused by work-related stress.

There is no common definition of stress in the literature. The fact that *stress* can refer to either exposure (stressors) or stress responses and reactions (physiological, behavioural, subjective etc.) can lead to confusion when using this term. Work-related stress has been defined as a pattern of stress responses/reactions (emotional, cognitive, behavioural and physiological) caused by the adverse aspects of work stressors (work content, organisation, environment) and is a state of high levels of arousal, distress and feelings of not coping [7]. The consequences of these reactions could then result in health problems (physical, mental or both) [7,8].

Considering the possible consequences of prolonged stress reactions, it is important to measure perceived stress in a valid and reliable way. A great deal of research has been done that involves monitoring work-related stressors, stress reactions and stress-related ill-health and there are many self-assessment checklists and questionnaires that have been designed for different purposes. One example is the Perceived Stress Scale, which is designed to assess whether situations in everyday life are perceived as stressful [9]. For work-related stress assessments, the Stress-Energy Questionnaire (SEQ) [10,11] has been used in many Scandinavian studies [12-18]. In a Swedish longitudinal cohort study of human service workers, the SEQ was used to identify individuals at risk of adverse health effects, i.e. individuals with a high level of stress and a low energy level at work [19,20].

The SEQ is often used in the same context as the Job Demand-Control model (JDC) [21]. The SEQ measures an emotional or affective stress response to a stressful situation at work whereas the JDC measures the stressors. The relationship between JDC and SEQ has been evaluated in a previous study [10]. It has been suggested that the stress response mediates the effects of work stressors on health. Hence, a work situation reported as stressful according to the JDC may not increase the risk of negative health effects if the worker does not regard the situation as stressful [13]. These results provide support for the usefulness of the SEQ in stress studies.

The usefulness of multi-item questionnaires also depends on the validity, which is a key quality component. Validation of self-assessed questionnaires is an ongoing process and involves accumulating evidence to provide a scientific basis for supporting study-specific purposes [22,23]. Responses to a multi-item instrument that are assessed on a rating scale produce ordinal data. The standard procedure for handling the rating scale data is numerical coding of responses with sequential numbers in an attempt to assert the severity of the trait being measured. A global mean score based on these numbers is calculated to represent the latent dimension being measured. However, due to the non-metric properties of the ordinal data, this procedure is not valid and should not be taken at face value. An important part of the validation process is the adequacy of the scaling of scores, which can be checked by employing modern psychometric techniques.

The content validation of the SEQ carried out previously by Kjellberg [10] also applies to our study. The relationship to other variables, e.g. between the JDC and the SEQ, was also investigated. Some indications of the differences in the use of the SEQ items by women and men are seen in a previous explorative study [24]. However, to our knowledge no analysis of the psychometric properties of the scale using modern analytical techniques has been published to verify the use of the global stress and energy scores. Consequently, the aim of this paper is to examine the internal construct validity of the SEQ in a working population using the Rasch analysis. This allows for formal testing for unidimensionality, which is a requirement for the construction of valid global scores. Additional measurement issues, such as category ordering (whether or not the category ordering of the items is working as expected) and potential differences between how women and men use the SEQ, termed differential item functioning (DIF), will also be evaluated.

## Methods

### Study design and population

The data for the present study is baseline data from a longitudinal cohort study where the aim was to evaluate psychosocial working conditions, stress, health and well-being among employees in two human service organisations in Western Sweden. The data was collected in 2004 through a postal questionnaire sent to a random sample (n = 5,300) of 48,600 employees of the Region Västra Götaland, a large public healthcare organisation, and a random sample (n = 700) of 2,200 social insurance office workers in the same geographical area. An inclusion criterion of at least one year of employment (at least 50% of full-time) was applied. Two reminders were sent to non-responders. Written informed consent for participation in the study was obtained from participants. The study was approved by the Regional Ethical Review Board in Gothenburg, Sweden, and it was conducted in accordance with the 1964 Declaration of Helsinki. The total response rate was 62%. Due to the selection criteria, the participants were mainly employed in the healthcare sector (86%). The three most common professions were nurse, assistant nurse and physician and the mean age was 48 years. Further demographic and study-specific details are available in published studies [19,25,26].

In this study, 2,817 individuals who responded to the SEQ items were available for analysis (2,378 women, 439 men). For evaluation of DIF, it is recommended that the groups compared are of approximately equal size, which ensures that if there is DIF, the results will not be dominated by the group that has the largest sample size [27,28]. Consequently, to achieve a balanced data set in terms of gender, approximately 20% of the women were sampled randomly from the original data set. The final study population thus comprised 441 women and 439 men. This sub-sample and the total sample were comparable in terms of age and profession.

### Measures

The SEQ is an adjective checklist with two dimensions – stress and energy – hypothesised to describe two critical aspects of mood at work. The original overall question to be answered through the checklist is: *“How do you usually feel at the end of a normal working day?”* In a modified version used in this study, the time perspective was changed to “*during the past week”*. Based on the theory of allostatic overload [29], we postulated that the dominant level of arousal during the past week rather than at the end of a working day would be more closely related to long-term stress exposure.

The SEQ is based on the circumplex model of affect proposed by Russell [30]. According to this theory, stress and energy represent bipolar dimensions, giving two total scores, one for the stress dimension and one for the energy dimension. Hence, the stress dimension ranges from positively evaluated low activation to negatively evaluated high activation. The energy dimension ranges from negatively loaded low activation to positively loaded high activation. Each dimension is operationalised using three positively oriented items (stress: *rested, relaxed, calm*; energy: *active, energetic, focused*) and three negatively oriented items (stress: *tense, stressed, pressured*; energy: *dull, inefficient, passive*). The response alternatives are: *not at all*, *hardly*, *somewhat, fairly, much* and *very much.* The interpretation of response categories goes in opposite directions for positive and negative items. For positively loaded items, *very much* implies the lowest stress level and the highest energy level (the most favourable response), while *not at all* is the least favourable response. The opposite is true for negatively loaded items. Response categories are coded numerically (0–5). The numerical coding of positively loaded stress items and negatively loaded energy items were reversed before calculating a total mean score for stress and energy respectively.

### Data analysis

The Rasch model, named after a Danish mathematician [31], is based on a latent trait theory and falls into the modern psychometric approach category. The model is intended for the development and evaluation of multi-item instruments. The Rasch model operationalises the axioms of additive conjoint measurements, which are the requirements for the measurement construction [32-35]. The SEQ data were fitted to the Rasch measurement model using the unrestricted or partial credit model for polytomous cases, which allows the distances between thresholds to vary across the items [36,37]. A threshold is the point between any two adjacent categories in which the probability of either response is equally likely. Data were fitted to the Rasch model using the RUMM2030 software [27]. Stress and energy dimensions were analysed separately as one of the assumptions for the Rasch analysis is unidimensionality.

The aim of the Rasch analysis is to see how well the observed data satisfy the model expectations. The Rasch analysis process involves testing a series of assumptions, including stochastic ordering of items (monotonicity), unidimensionality, local independency and principle of invariance [38]. The adequacy of fit is evaluated using multiple fit statistics and their ideal values are shown at the bottom of summary fit Table 1 [39].

**Table 1:** **Fit to the Rasch model**

	**Item residual**		**Person residual**		**Chi square**		**Unidemensionality**	
**Analysis name**	**Mean**	**SD**	**Mean**	**SD**	**Value**	**p**	**PSI**	**Test % (95% CI)**
1 Stress, 6 items	0.48	1.30	−049	1.19	52.79	0.52	0.92	10.5 (9.1;12.0)
2 Stress 2 testlets	0.31	0.56	−0.62	1.03	13.57	0.75	0.87	4.4 (3.0;5.9)
3 Energy, 6 items	−0.008	2.05	−0.43	1.09	70.61	0.06	0.80	8.4 (7.0;9.9)
4 Energy, 4 items	−0.31	2.35	−0.42	0.89	47.76	0.04	0.75	6.1 (4.6;7.5)
5 Energy re-scoring	0.03	2.07	−0.42	1.09	72.17	0.05	0.80	8.3 (6.9;9.8)
6 Energy DIFsplit	−0.13	1.83	−0.42	1.08	74.73	0.15	0.80	
7 Energy 2 testlets	0.13	0.33	−0.49	0.83	23.20	0.18	0.70	3.3 (2.2;5.1)
***Ideal values***	***0.0***	***<1.4***	***0.0***	***<1.4***		***>0.05***	***>0.7***	***(LCI <5%)***

Stochastic ordering of items is evaluated through the fit of data to the model. The response structure required by the Rasch model is a stochastically consistent item order, i.e. a probabilistic Guttman pattern [40]. This means that persons who experience higher stress or energy levels are expected to get higher scores, whereas persons with lower stress or energy levels are expected to get lower scores. The intended increasing level of stress and energy across the response categories for each item needs to be reflected in the observed data. The Rasch analysis can be used to see if items are categorised correctly and threshold ordering was considered for this purpose. In the case of disordered thresholds, the items can be rescored by collapsing the categories [38]. The disordering of the thresholds can be viewed graphically by plotting category probability curves.

The invariance criterion implies that the items need to work in the same way (invariantly) across the whole continuum of the latent trait for all individuals. In that case, the relative position of the items, i.e. the ratio between the location values of any two items, must be constant along the trait. Also given the same level of the latent trait (stress or energy), the scale should function in the same way for all comparable groups (e.g. gender). This is commonly known as differential item functioning (DIF). In the presence of DIF, women and men would score differently for a specific item, given the same level of stress or energy.

Three overall fit statistics were considered*.* The item-trait interaction is the χ^2^ statistic and reflects the property of invariance across the trait. A significant value indicates that the hierarchical ordering of the items varies across the stress or energy trait. The other two statistics are the item-person interaction statistics, transformed to approximate a standardised normal distribution. In the case of fit, the expected values are a mean of 0 and a standard deviation (SD) of 1. In addition to the overall fit, the individual item and person fit were also considered, both as residuals and as a χ^2^ statistic. A perfect fit is indicated by a standardised fit residual value of ±2.5 and a non-significant χ^2^.

In this study, DIF for gender was tested by conducting ANOVA of standardised residuals, which enables separate estimations of misfit along the latent trait, uniform and non-uniform DIF. In the case of uniform DIF, there is a consistent systematic difference in the response to an item across the whole range of the latent trait [41]. Non-uniform DIF means that the magnitude of DIF is not constant across the trait. Detection of DIF can be dealt with by splitting a misfitted item into two items, one item for women, with missing values for men, and the other for men, leaving women with non-responses [38]. In order to understand the nature and magnitude of DIF, the initial and resolved analysis can be compared in terms of parameter estimates, given fit to the model [28,42].

Local dependency is manifested in two ways – through response dependency and trait dependency, both analysed by means of residual correlations [43]. The response dependency is where items are linked in a way that the response to one item will depend on the response to another item. The presence of response dependency inflates reliability, compromises parameter estimation and can be detected through the correlation of residuals [44], which in the current analysis is a value of 0.2 above the average residual correlation. The trait dependency is a violation of unidimensionality.

Unidimensionality is a basic prerequisite for combining any set of items into a total score. Smith’s test of unidimensionality is implemented in RUMM2030 [45]. For this test, items loading positively and negatively on the first principal component of the residuals are used to make an independent person estimate (in this case, stress and energy), and are then contrasted through a series of independent t-tests [45]. Less than 5% of such tests would support the unidimensionality of the scale. A 95% binomial confidence interval of proportions can be used to show that the lower limit of the observed proportion is below the 5% level [45]. If detected, the local dependency can be accommodated by combining locally dependent items into a ‘super item’ or testlets [46].

In Rasch analyses, both persons and items are calibrated on the logit scale and this enables an evaluation to be made of how well-targeted the items are for the persons in the sample. This can be assessed by comparing the observed mean location score for persons with that of the value of the items, which is set at zero. For a well-targeted instrument, the mean location for persons would be around zero. A positive mean value for persons would indicate that the sample as a whole was located at a higher level of stress or energy that the average of the scale. The reverse is true for negative values. This is presented graphically in the form of a person-item distribution graph. Reliability is reported as a Person Separation Index (PSI), interpreted in a similar way to the Cronbach’s alpha. Values of 0.7 and 0.9 are indicative of sufficient reliability for group and individual use respectively [47].

## Results

### Stress dimension

An initial analysis of the six stress items was made and a summary fit statistics are shown in Table 1 (Analysis 1). The fit to the model was relatively good, with a non-significant χ^2^ statistic and high reliability (PSI). In addition, item and person residual fit also indicated a good fit. No DIF by gender was observed. As regards individual item fit, all items had standardised residual fit values within the predefined range of ±2.5 and a non-significant χ^2^. None of the items had disordered thresholds.

**Figure 1:**
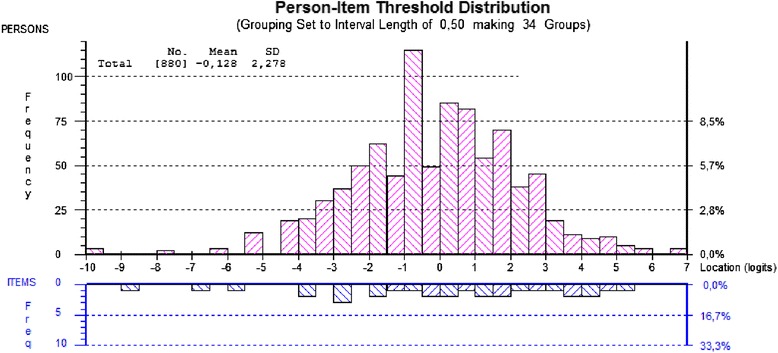
**Person item distribution graph for the stress scale of the Stress-energy questionnaire.**

Test of unidimensionality revealed certain problems. An examination of the residual correlation matrix gave indication of the response dependency between the following pairs of items: *rested* and *relaxed*, *relaxed* and *calm*, *stressed* and *pressured*. The assumption of trait dependency was compromised as correlated residuals clustered within the two groups of items: positively loaded items (*rested*, *relaxed*, and *calm*) and negatively loaded items (*stressed*, *pressured*, and *tense*). Consequently, these items were grouped as testlets and additional analysis was carried out, resulting in a better fit to the model (Table 2, Analysis 2). Some reduction in reliability was observed as a consequence of accommodating local dependency through testlets. The PSI decreased to 0.87, which is between the two predefined values of 0.7 and 0.9. A test of unidimensionality also showed improved fit, as the lower limit of the 95% CI no longer included 5%. The distribution of items and persons on a logit scale is shown in Figure 1, indicating satisfactory targeting. Given the fit to the model, a transformation of the ordinal raw score to an interval score was conducted and presented in Table 2.

**Table 2:** **Stress dimension of the Stress-Energy Questionnaire, transformation of raw mean score to metric score**

**Raw score**	**Stress**
0	0
0.17	0.61
0.33	1.05
0.50	1.38
0.67	1.62
0.83	1.82
1	1.99
1.17	2.14
1.33	2.28
1.50	2.41
1.67	2.54
1.83	2.66
2	2.77
2.17	2.87
2.33	2.97
2.50	3.07
2.67	3.16
2.83	3.25
3	3.34
3.17	3.43
3.33	3.52
3.50	3.61
3.67	3.70
3.83	3.80
4	3.90
4.17	4.01
4.33	4.13
4.50	4.26
4.67	4.43
4.83	4.66
5	5

### Energy dimension

**Figure 2:**
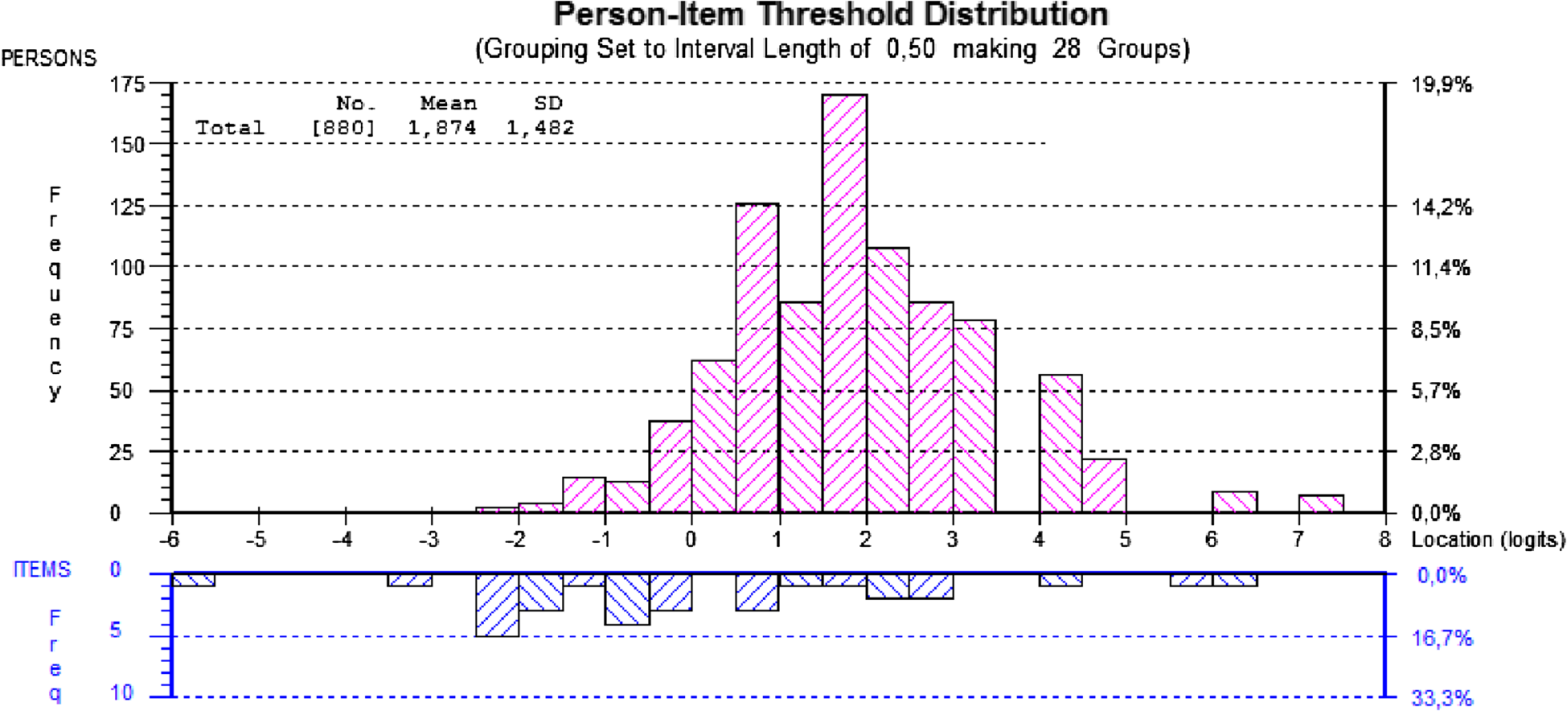
**Person item distribution graph for the energy scale of the Stress-energy questionnaire.**

Fitting the energy items to the Rasch model revealed a non-optimal fit to the model expectations (Table 1, Analysis 3). The item residual SD was much higher than the ideal value of 1.4. Out-of-bound fit residuals for individual items were found for the items *inefficient* and *passive*, 3.50 and −2.53 respectively. However, these values were not statistically significant, after the Bonferroni adjustment. Moreover, a PSI of 0.8 was below the minimum acceptable value for individual use (0.9) in a clinical setting but was still above the acceptable level for use in group comparisons (0.7). The targeting of the items and persons along the energy trait was not good (Figure 2). The majority of the respondents reported high energy levels, i.e. assessment of high energy categories for most of the items. The category frequencies are shown in Table 3. Additional adjustments to the energy dimension were done by deleting the items with high residual values (*inefficient* and *passive*), however this did not improve the fit to the model (Table 1, analysis 4).

**Table 3:** **Category response frequencies, Energy items, n (%)**

**Items**	**Cat 1**	**Cat 2**	**Cat 3**	**Cat 4**	**Cat 5**	**Cat 6**
**Active**	1 (<1)	11 (1)	55 (6)	263 (30)	463 (53)	87 (10)
**Energetic**	8 (1)	61 (7)	172 (20)	377 (43)	237 (27)	25 (3)
**Focused**	3 (<1)	16 (2)	86 (10)	407 (46)	334 (38)	34 (4)
**Dull**	1 (<1)	12 (1)	26 (3)	171 (19)	293 (33)	377 (43)
**Inefficient**	2 (<1)	18 (2)	46 (5)	200 (23)	353 (40)	261 (30)
**Passive**	0 (0)	6 (1)	29 (3)	105 (12)	355 (40)	385 (44)

Disordered thresholds were observed for the item *dull*. The ordering of the thresholds suggests problems discriminating the first three categories (diagram not shown). Additional analysis was performed by rescoring the item *dull* into five categories, i.e. by collapsing the second and third response categories. This solution produced ordered thresholds for all items. However, the fit to the model was not improved (Table 1, Analysis 5). The change in individual person location from this additional analysis (mean 1.90, SD 1.49) compared with those from the original analysis (mean 1.87, SD 1.48) was marginal (mean difference −0.03, 95% CI −0.17; 0.11). Consequently, the rescoring did not seem justified. Alternative rescoring procedures were also checked and did not result in ordered thresholds.

**Figure 3:**
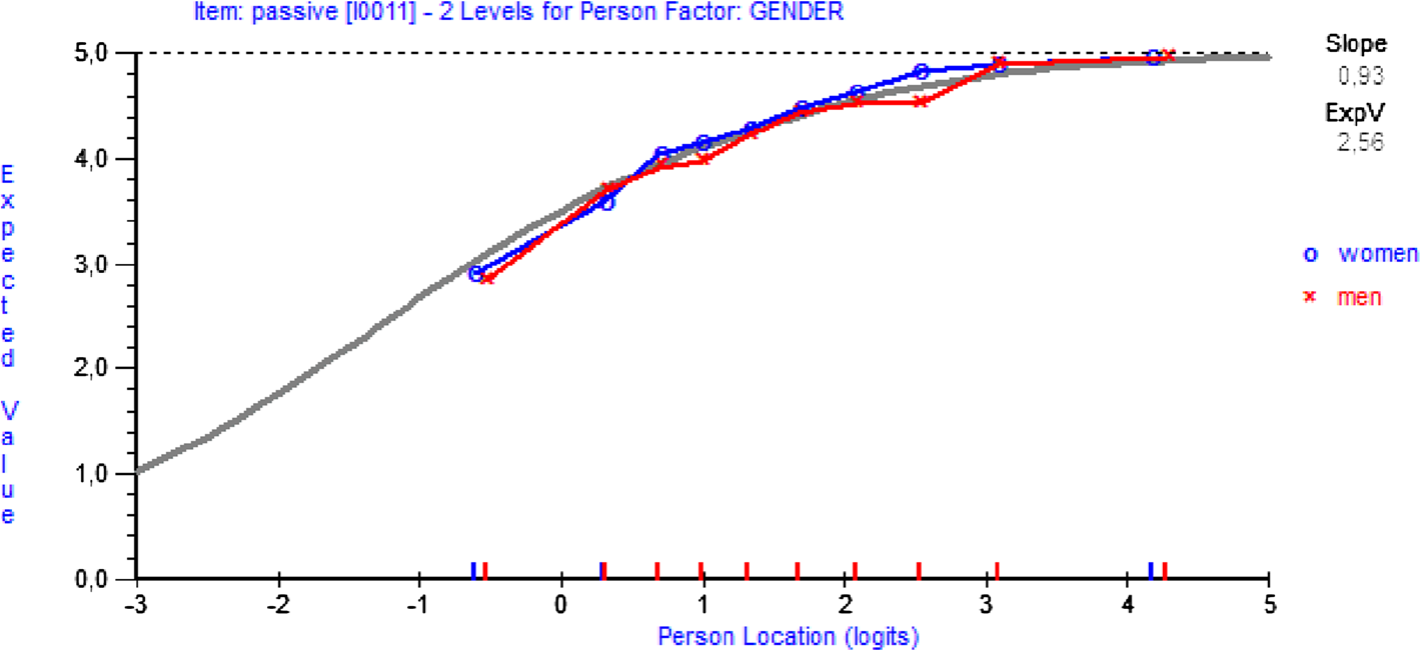
**Category probability curves item** ***passive.***

Analysis of variance based on standardised residuals indicated uniform DIF for gender only for the item *passive* (F = 9.63, df = 1,879, p = 0.002)*.* Given the same level of energy, women rated slightly higher for this item compared to men, as shown in Figure 3. The class interval in ANOVA was also significant (F = 3.06, df = 19, p = 0.99), indicating poor fit of this item to the model, as suggested previously by the out-of-bound residual value. There is no evidence of non-uniform DIF for any of the items. Consequently, additional analysis was done by splitting the item *passive* for women and men (Table 1, Analysis 6), showing almost no change in the fit to the model compared to the initial analysis.

Pairwise correlations between the energy items indicated the response dependency between the following pairs of items: *active* and energetic, *dull* and *passive*. Another source of misfit was that correlated fit residuals in the first principal component clustered into two groups, those of positively loaded items (*active*, *energetic*, *focused*) and negatively loaded items (*dull*, *passive*, *inefficient*). Given this observed local dependency, additional analysis was done by grouping the positively and negatively loaded items into two testlets (Table 1, Analysis 7). The fit to the model expectation was achieved. The PSI value decreased to the low but still acceptable level of 0.7.

## Discussion

Data from the working populations showed that the psychometric properties of the stress scale of the SEQ were satisfactory, having accommodated for local response dependency through the use of testlets. All stress items worked properly for this working population employed in Swedish human service organisations and for both women and men. The results suggest that the scale can be used for assessment of work-related stress as the total score reflects the scoring structure indicated by the developer. The raw mean score, which is ordinal, has been transformed to the interval scale latent estimate. We recommend the use of the transformed score in statistical analyses instead of the raw mean score, given no missing values on any of the items. However, the cut-off value, which indicates high and low levels of stress and thus identifies the risk groups for adverse health effects, needs to be re-determined, which is beyond the scope of this article.

The initial analysis energy scale did not show an equally good fit to the model. The items were not well targeted to the persons, with a skewness towards high energy. In other words, there was too little variation in the energy levels to be able to differentiate between the subjects. This could be a probable explanation for the low reliability (low PSI value) and poor match between persons and items (mistargeting), since the majority of the respondents endorsed high energy categories for most of the items. The PSI is an estimate of how well the scale can differentiate subjects on the measured variable, in this case energy. A low PSI (<0.7) indicates problems with reliability and a high value is needed (>0.9) for evaluations of individual persons. The PSI value decreases towards zero as the mismatch between the person and item distributions becomes more pronounced [27]. The robust estimation of the threshold parameters depends on having sufficient observations for each of the corresponding response categories [48]. One strength of this study is a relatively large sample size. Some limitations should be mentioned. The ideal scenario would be a well-targeted sample, which is not the case regarding the energy scale in the present data. Provided good targeting, the Rasch person estimates (logits), can be transformed to a convenient range for an easier interpretation. This was done for the stress scale and presented in Table 2. However, the lowest energy levels were not observed in our data. Consequently, the transformation table for the energy scale was not given. In order to improve the targeting, one possibility is to adjust the energy scale by including new items which would be better targeted to perceived energy levels in this type of highly performing working populations. Another possibility could be to test the energy scale in a more varied working population.

One reason for evaluating item performance is to examine whether the subjects use the response categories as intended. In other words, are increasing levels of stress and energy across the response categories for each item reflected in the observed data? Another important aspect is checking whether all responses, from *not at all* (implying the lowest energy level) to the highest energy level, *very much,* were used. Disordered thresholds were found for three lowest categories in the energy item *dull*. The issue of disordering can be resolved by collapsing the disordered thresholds. Consequently, the categories *much* and *fairly* for the item *dull* were collapsed and the results from the original analysis and this additional analysis were compared. Although this solution resulted in ordered thresholds for all items, we did not see a substantial improvement in the fit to the model. Hence, the disordered thresholds for this item could perhaps be explained by not having enough respondents in low energy categories and not by the respondents having problems discriminating between the categories. This explanation is also supported in the literature, since it has been suggested that estimation of the threshold parameters could be problematic when there are no or very few observations for certain categories and the lack of data may result in disordered threshold estimates for categories within the region [27,48]. Another plausible explanation for the disordered thresholds in the item *dull* may be the influence of social acceptance in the answers. Do we really admit to being *very much passive* or *not at all active* at work?

Potential DIF for gender was found for the item *passive*, which was uniform. This item was also identified as problematic in a previous study [24] where women reported *not at all passive* more frequently than men, whereas men utilized *hardly passive* more than women (although not adjusted for the total energy levels). The same pattern is seen in this study. Given the same energy level, men rated lower response categories than women. Splitting the item for women and men could be a way to resolve DIF and this is something we tested. A consequence of handling DIF in this way is that the resolved item has different parameter estimates for each group and is no longer invariant across the groups. However, the fit to the model was not obtained. Consequently, our results do not justify resolving DIF by splitting this item, although attention should be paid to this in future studies.

Two misfitted items, *inefficient* and *passive*, were identified by means of out-of-bound residuals (although statistically non-significant). A very high negative value implying overfit, i.e. redundancy of an item and usually a result of violation of local independency, is of less concern than underdiscrimination (indicated by very high residual values) [43,49]. The residual value for the item *passive* was slightly below the limit and the problem is resolved by forming the two testlets of positively and negatively loaded items. Underdiscrimination, usually indicated by high positive residual values, might suggest violation of unidimensionality [43]. This was also resolved by fitting the model to the testlets. Hence, the energy dimension also fitted the expectations of the Rasch model in the final analysis.

Local dependency problems were noted for both stress and energy dimensions, as in both scales fit residuals of positively and negatively oriented items clustered together. The results suggest that these items cluster in groups and measure opposite directions on the same trait. This poses a problem from the point of measurement. However, this result is also in line with the theoretical foundation of the model used for the development of the SEQ, where positively and negatively loaded adjectives are used to capture the differences in how people express their perceptions of mood. The problem was solved by forming of testlets, a solution useful a measurement point, still satisfying the underlying theory and keeping all the items in the SEQ. As described by Kjellberg et al., positively and negatively loaded items are seen as bipolar dimensions within each dimension [10]. Local dependence for both scales was addressed using testlets.

## Conclusion

The stress scale of the SEQ satisfies the measurement criteria defined by the Rasch analysis and provides a useful tool for work-related stress assessment, both for individual use and on a group level. Energy assessments also confirmed the fit to the model but need to be evaluated further in different settings and populations. In this population the energy scale is suitable for group assessments only.

## Abbreviations

ANOVA: Analysis of variance
DIF: Differential item functioning
JDC: Job Demand-Control model
PSI: Person Separation Index
SD: Standard deviation
SEQ: Stress-Energy Questionnaire
